# 微波热疗协同吉西他滨抑制人肺鳞癌细胞增殖及诱导凋亡机制研究

**DOI:** 10.3779/j.issn.1009-3419.2018.11.01

**Published:** 2018-11-20

**Authors:** 洋 杨, 妍妍 赵, 胜林 马, 道科 杨

**Affiliations:** 1 450002 郑州，郑州大学第一附属医院放疗科 Department of Radiation Oncology, the First Affiliated Hospital of Zhengzhou University, Zhengzhou 450002, China; 2 310006 杭州，杭州市第一人民医院，浙江大学医学院附属杭州市第一人民医院转化医学中心 Affiliated Hangzhou First People' s Hospital, Zhejiang University School of Medicine, Hangzhou 310006, China

**Keywords:** 微波热疗, 吉西他滨, 肺鳞癌, 增殖, 凋亡, Microwave hyperthermia, Gemcitabine, Lung squamous cell carcinoma, Proliferation, Apoptosis

## Abstract

**背景与目的:**

肺癌是全球发病率及死亡率最高的恶性肿瘤之一，寻找有效的抗肿瘤方法显得很重要，微波热疗作为一种新的治疗技术越来越受到重视。本研究旨在探讨微波热疗联合吉西他滨对人肺鳞癌细胞NCI-H1703和NCI-H2170体外增殖的影响及诱导凋亡的机制。

**方法:**

CCK-8法分别检测微波热疗、吉西他滨对细胞增殖影响以及微波热疗与吉西他滨不同序贯方式对细胞增殖的影响；克隆形成实验观察阴性对照组、微波热疗组、吉西他滨组及热化联合组对细胞克隆形成的影响；流式细胞术检测不同处理组细胞的总凋亡率；Caspase-3、Caspase-8活性检测试验检测各组细胞Caspase-3、Caspase-8酶活性；CCK-8法检测AC-DEVD(Caspase-3抑制剂)对细胞增殖的影响；Western blot检测微波热疗和吉西他滨对肺鳞癌细胞凋亡相关蛋白的表达。

**结果:**

微波热疗对肺鳞癌有增殖抑制作用；吉西他滨对两株细胞的IC_50_值分别为8.89 μmol/L和44.18 μmol/L；先化疗后微波热疗对两株肺鳞癌细胞有协同作用且可明显抑制细胞的克隆形成(*P* < 0.001)、促进细胞凋亡(*P* < 0.001)以及显著提高Caspase-3酶活性(*P* < 0.001)，但对Caspase-8酶活性影响不大(*P* > 0.05)；加入AC-DEVD(Caspase-3抑制剂)后，热化联合组细胞增殖率提高(*P* < 0.001)；Western blot检测显示微波热疗联合吉西他滨可上调p53、Caspase-3、Cleaved-Caspase-3、Cleaved-PARP以及Bax蛋白的表达。

**结论:**

先吉西他滨后微波热疗可协同抑制人肺鳞癌细胞的体外增殖以及促进凋亡，其机制可能是通过活化p53、切割PARP蛋白诱导癌细胞发生Caspase-3依赖性凋亡，从而发挥其协同抑制癌细胞增殖的作用。

## Abstract (en)

**Background and objective:**

Lung cancer is one of the highest morbidity and mortality in the world and it is very important to find an effective anti-tumor method. Microwave hyperthermia, a new treatment technology, has been getting more and more attention. This study was designed to investigate the effects of microwave hyperthermia combined with gemcitabine on the proliferation and apoptosis of human lung squamous cell carcinoma (NCI-H1703 and NCI-H2170) *in vitro*.

**Methods:**

The proliferation of cells treated with microwave hyperthermia, the effect of gemcitabine on cell proliferation and the proliferation of cells treated with different methods of microwave hyperthermia and gemcitabine were detected by CCK-8 assay. Colony formation assay was used to measure the colony formation of human lung squamous cell carcinoma cells. Flow cytometry assay was used to detect the total apoptosis rates of the treated cells. Caspase-3, Caspase-8 activity assay was used to detect the activity of Caspase-3, Caspase-8 enzyme in each group of cells. CCK-8 assay was used to detect the effect of control group, AC-DEVD (Caspase-3 inhibitor) group, thermalization combined group, and thermal AC-DEVD combined group on cell proliferation. The levels of p53, Caspase-3, Cleaved-Caspase-3, PARP, Bax and BCL-2 protein expression were detected using Western blot assay.

**Results:**

Our results demonstrated that microwave hyperthermia inhibited the proliferation of lung squamous cell carcinoma. The IC_50_ values of gemcitabine for the two cells were 8.89 μmol/L and 44.18 μmol/L, respectively. The first chemotherapy after microwave hyperthermia has synergistic effect on the two lung squamous cell carcinoma cells and can significantly inhibit the cell clone formation (*P* < 0.001), promote cell apoptosis (*P* < 0.001) and increase Caspase-3 enzyme activity (*P* < 0.001). However, it has no effect on Caspase-8 enzyme activity (*P* > 0.05). Furthermore, Western blot analysis showed that microwave hyperthermia combined with gemcitabine could up-regulate the p53, Caspase-3, Cleaved-Caspase-3, Cleaved-PARP and Bax protein expression.

**Conclusion:**

Microwave hyperthermia combined with gemcitabine remarkably inhibit the proliferation and induce apoptosis of human lung squamous cell carcinoma *in vitro*. This effect may be associated with the activation of p53, cleavage of PARP protein, and induced the Caspase-3 dependent apoptosis.

肺癌是世界上癌症相关死亡的主要原因，非小细胞肺癌(non-small cell lung cancer, NSCLC)约占肺癌的80%-85%^[1]^，肺鳞癌作为NSCLC常见的类型之一，经过手术、放化疗等治疗后，其5年生存率仍低于15%^[2]^。最新美国国立综合癌症网络(National Comprehensive Cancer Network, NCCN)指南提出肺鳞癌的一线治疗方案仍然是吉西他滨与铂类药物联用，然而大多数患者接受了一线治疗方案后常出现耐药现象，进而不可避免地使肿瘤发生进展^[3]^。对于一线治疗失败的患者，NCCN指南推荐多西他赛作为肺鳞癌的二线治疗，但由于多西他赛毒性反应显著，临床用药常常受到限制^[4, 5]^。尽管分子靶向药物和免疫治疗在肺腺癌的治疗中取得了重大作用，但对于肺鳞癌而言，目前尚无明确的靶向药物治疗。因此在新的有效药物出现之前，如何提高肺鳞癌细胞对吉西他滨的敏感性或者发现新的联合疗法对临床上提高患者的有效生存时间和生活质量显得尤为重要。

近年来，微波热疗因其安全有效、毒副作用小、患者耐受性好等优点在临床上被广泛应用，如：肝癌、乳腺癌、膀胱癌、肺癌等^[6-11]^，但单纯的微波热疗往往难以取得显著的治疗效果，其常与化疗药物联合应用以提高化疗疗效。临床上，微波热疗联合吉西他滨治疗肺鳞癌虽已取得显著疗效，但具体的分子机制尚不明确。因此，本课题组自主研发了与临床微波热疗原理一致的供基础研究使用的微波热疗仪，以探索与吉西他滨药物联合使用对肺鳞癌细胞增殖抑制的影响及诱导凋亡的机制，从而为肺鳞癌的精准治疗提供新的思路，为改善患者的不良预后探索新的方案。

## 1 材料和方法

### 1.1 主要仪器与试剂

微波热疗仪(专利号：CN204824903U)；FACSCanto Ⅱ型流式细胞仪(美国BD公司)；SpectraMaxM3型全波长多功能酶标仪(美国Molecular Devices公司)；ChemiDoc XRS+型凝胶成像系统(美国Bio-Rad公司)。RPMI-1640培养基、胎牛血清(美国Gibco公司)；胰蛋白酶-EDTA消化液(0.25%)、吉西他滨(法国礼来公司)、CCK-8(美国MCE公司)；荧光素异硫氰酸(FITC)标记的膜联蛋白Ⅴ(Annexin Ⅴ-FITC)/碘化丙啶(PI)凋亡检测试剂盒(美国BD公司)；Caspase-3和Caspase-8活性检测试剂盒(中国碧云天)；Caspase-3抑制剂(AC-DEVD)(中国碧云天)；兔抗人Bcl-2抗体(美国CST公司)、兔抗人Bax抗体(美国CST公司)、兔抗人p53抗体(美国CST公司)、兔抗人Caspase-3抗体(美国CST公司)、兔抗人Cleaved-Caspase-3抗体(美国CST公司)、兔抗人PARP抗体(美国CST公司)和鼠抗人β-actin抗体(美国Abcam公司)；辣根过氧化酶(HRP)标记的二抗(Abbkine公司)等。

### 1.2 细胞培养

人肺鳞癌细胞株NCI-H1703及NCI-H2170均购于中国科学院上海生物化学与细胞生物学研究所细胞库，两株细胞以含10%灭活胎牛血清的RPMI-1640培养液于37 ℃、5%CO_2_细胞培养箱内培养。当细胞铺展覆盖培养瓶底部面积80%时，以0.25%胰蛋白酶消化，待镜下观察收缩至圆形且脱落时，加入RPMI-1640培养液终止消化，1, 000 rpm 5 min离心后，按适当比例(1:3)进行传代，取对数生长期的细胞进行实验。

### 1.3 微波热疗仪输出功率及温度的控制说明

本课题组研发的应用于肿瘤基础研究的微波热疗仪(专利号：CN204824903 U)是由一个433 MHz的微波源、微波辐射器、光纤温度计探头、温度自动控制系统以及数据记录和显示系统(计算机)组成。在微波热疗期间，通过两个光纤温度计探头测量培养细胞的温度以及周围循环水的温度。此温度计探头测温精度在±0.2 ℃以内，可以避免微波对测温的干扰。温度自动控制系统可以通过增加输出功率使温度保持在预先设定的恒定值(±0.2 ℃以内)，微波输出功率的范围在50 w-200 w。

### 1.4 CCK-8法检测微波热疗和吉西他滨对人肺鳞癌细胞株NCI-H1703和NCI-H2170细胞增殖的影响

微波热疗温度设置对照组(不加任何热处理仅放置于37 ℃ 5%CO_2_孵箱内)、40 ℃、41 ℃、42 ℃、43 ℃共5组，作用时间分别为30 min、60 min、90 min；每组设置5个复孔：取对数生长期的人肺鳞癌细胞NCI-H1703和NCI-H2170，0.25%胰酶消化洗涤后用完全培养液配成单细胞悬液，以每孔1×10^4^个细胞接种至96孔板中，每孔100 μL，37 ℃、5%CO_2_中培养24 h后对照组更换培养液继续37 ℃、5%CO_2_中培养，微波热疗组(40 ℃、41 ℃、42 ℃及43 ℃)更换培养液后分别给予不同温度微波热疗处理30 min、60 min、90 min后，继续培养24 h后，弃掉培养液，然后每孔加入含10%CCK-8的RPMI-1640培养液100 μL，37 ℃、5%CO_2_培养箱中再继续培养1 h-3 h，将培养板置于酶标仪上，以450 nm为测定波长，测定各孔的吸光度OD值并计算细胞增殖率，以80%增殖率左右的处理组作为后续实验的微波热疗组(MW)。

吉西他滨设100 μmol/L、50 μmol/L、25 μmol/L、12.5 μmol/L、6.25 μmol/L、3.125 μmol/L、1.56 μmol/L、0 μmol/L共8个浓度组，每组设置5个复孔，以CCK-8法(方法同1.5)测定细胞增殖率，计算药物浓度的IC_50_以及70%左右增殖率的药物浓度作为后续实验的吉西他滨组(Gem)。

微波热疗和吉西他滨以不同序贯方式进行处理：热化同时进行组(加药后立即进行热疗，Gem and MW)，先热疗后化疗组(微波热疗后间隔24 h再化疗，MW then Gem)以及先化疗后热疗组(化疗24 h PBS洗净后微波热疗，Gem then MW)。以CCK-8法测定各组细胞的增殖情况(方法同1.5)，根据Veleriote法^[11]^判定热化疗联合的相互作用类型。协同作用：[C] < [E]；相加作用：[C]=[E]；次加作用：[E] < [C] < [H]或[E] < [C] < [D]；干扰作用：[D] < [C] < [H]。其中：[H]为热疗组的细胞存活率，[D]为化疗组的细胞存活率，[C]为热化疗组的细胞存活率，[E]为热化疗联合的预估细胞存活率，[E]=[H]×[D]。并选出最佳序贯方法用于后续研究。

细胞增殖率=实验组OD均值/对照组OD均值×100%。

### 1.5 克隆形成实验

收集对数生长期的NCI-H1703和NCI-H2170细胞，消化成单个细胞悬液后，以1×10^3^个细胞/孔的密度接种于4组6孔板中(对照组、微波热疗组、吉西他滨组以及热化联合组)，置于细胞培养箱中培养24 h后，分别进行相应处理后置于培养箱中培养2周。2周后取出，弃去原培养液，以磷酸盐缓冲液(PBS)洗2遍后用0.1%的甲醛固定20 min，再用结晶紫染液染色20 min。PBS洗干净后晾干，以 > 50个细胞为1个克隆，显微镜下拍照计算细胞克隆形成数目。试验重复3次。

### 1.6 倒置显微镜观察各组细胞形态变化

收集对数生长期的NCI-H1703和NCI-H2170细胞，消化成单个细胞悬液后，以1×10^6^个细胞/孔的密度接种于4组6孔板中(对照组、微波热疗组、吉西他滨组以及热化联合组)，置于细胞培养箱中培养24 h后，分别进行相应处理后，倒置显微镜下观察并拍照记录。试验重复3次。

### 1.7 AnnexinⅤ-FITC/双染法流式细胞术检测细胞的凋亡

收集对数生长期的NCI-H1703和NCI-H2170细胞，消化成单个细胞悬液后以5×10^5^个细胞/孔的密度接种于4组6孔板中(对照组、微波热疗组、吉西他滨组以及热化联合组)，将细胞置于培养箱中培养24 h后，分别进行相应处理，24 h后收集上清培养液，用胰酶消化细胞并收集。之后加入300 μL的Annexin Ⅴ-FITC凋亡检测试剂盒中的缓冲液、5 μL的Annexin Ⅴ-FITC染液，轻轻混匀后，于2 ℃-8 ℃避光条件下培养15 min。再加入5 μL的PI，轻轻混匀，于2 ℃-8 ℃避光条件下培养5 min后，流式细胞仪检测，计算细胞总凋亡率(%)。试验重复3次。

细胞总凋亡率=早期凋亡率(Q2)+晚期凋亡率(Q4)。

### 1.8 Caspase-3、Caspase-8活性检测实验检测细胞Caspase-3、Caspase-8酶活性

收集对数生长期的NCI-H1703和NCI-H2170细胞，消化成单个细胞悬液后以5×10^5^个细胞/孔的密度接种于4组6孔板中(阴性对照组、微波热疗组、吉西他滨组以及热化联合组)，将细胞置于培养箱中培养24 h后，分别进行相应处理，24 h后收集上清培养液，用胰酶消化细胞并收集。之后按试剂盒说明书进行处理，然后将培养板置于酶标仪上，以405 nm为测定波长，测定各孔的吸光度OD值，通过Bradford法测定各组细胞的蛋白浓度，然后与ρNA标准曲线对比计算出各组细胞中催化产生的ρNA。试验重复3次。

### 1.9 CCK-8法检测AC-DEVD(Caspase-3抑制剂)对细胞增殖的影响

收集对数生长期的NCI-H1703和NCI-H2170细胞，消化成单个细胞悬液后以1×10^4^个细胞/孔的密度接种于4组96孔板中(阴性对照组、AC-DEVD组、热化联合组以及AC-DEVD+热化联合组)，将细胞置于培养箱中培养24 h后，分别进行相应处理后以CCK-8法(方法同1.5)测定各组细胞增殖率。试验重复3次。

### 1.10 Western blot检测细胞凋亡相关蛋白的表达

收集对数生长期的NCI-H1703和NCI-H2170细胞，消化成单个细胞悬液后以5×10^5^个细胞/孔的密度接种于4组6孔板中(阴性对照组、微波热疗组、吉西他滨组以及热化联合组)，将细胞置于培养箱中培养24 h后分别进行相应处理，24 h后提取细胞总蛋白，经BCA法测定蛋白浓度，取20 μL总蛋白上样，以β-actin为内参进行十二烷基磺酸钠-聚丙烯酰胺凝胶电泳(SDS-PAGE)，并转移至聚偏二氟乙烯膜(PVDF)上，5%脱脂奶粉室温封闭2 h，加入1:1, 000稀释的一抗于4 ℃孵育过夜，用缓冲液TBST洗膜3次，每次10 min。然后加入HRP标记的二抗(1:5, 000)，于室温条件下孵育2 h，再用缓冲液TBST洗膜3次，每次5 min。用超敏化学发光试剂显影检测，应用化学发光成像仪进行图像采集，以目标蛋白条带的灰度值与内参β-actin条带灰度值的比值表示目标蛋白的相对表达量。试验重复3次。

### 1.11 统计学方法

采用SPSS 17.0和GraphPad Prism 7.0软件进行数据分析及绘图。计量资料以均数±标准差(Mean±SD)表示，采用单因素方差分析和*t*检验进行组间比较。*P* < 0.05表示差异有统计学意义。

## 2 结果

### 2.1 微波热疗联合吉西他滨抑制人肺鳞癌细胞的增殖

首先，为了选择合适的微波热疗条件，我们将微波热疗温度设置对照组(37 ℃)、40 ℃、41 ℃、42 ℃、43 ℃共5组，作用时间分别为30 min、60 min、90 min，采用CCK-8法检测各组细胞的增殖率。其结果显示：与对照组相比，43 ℃ 30 min微波热疗组细胞增殖率稍有下降(*P* < 0.05)；41 ℃、42 ℃以及43 ℃ 60 min微波热疗组细胞增殖率都有所下降(*P* < 0.05或*P* < 0.001)；40 ℃、41 ℃、42 ℃以及43 ℃ 90 min微波热疗组细胞增殖率下降(*P* < 0.001或*P* < 0.01)；这表明随着温度的升高，作用时间的延长，两株细胞的增殖率也随之下降(图 1A-B)，后续实验选择42 ℃作用60 min的微波热疗组处理两株细胞；接着CCK-8结果显示吉西他滨对两株肺鳞癌细胞(NCI-H2170和NCI-H1703)的增殖率呈明显的浓度依赖性降低，吉西他滨作用于两株肺鳞癌细胞24 h后的IC_50_分别为8.89 μmol/L和44.18 μmol/L，后续实验选择5 μmol/L处理两株细胞(图 1C-D)；最后，与单独吉西他滨组对比，三种热化序贯方式的增殖率都有所下降(*P* < 0.05或*P* < 0.01或*P* < 0.001)，差异有统计学意义(图 1E-F)。根据Veleriote法^[11]^判断出热化同时组和先热疗后化疗组为次加作用；而先化疗后微波热疗组具有协同作用(表 1和表 2)。后续实验热化联合组采用先化疗后热疗的序贯方式。

**1:**
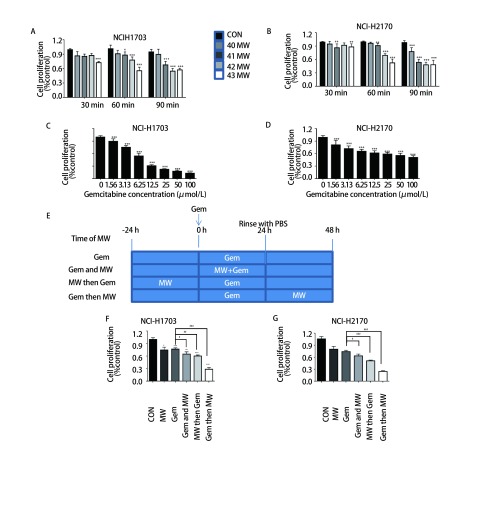
微波热疗和吉西他滨对两株肺鳞癌细胞的增殖影响。A、B：不同温度微波热疗作用不同时间对肺鳞癌细胞增殖影响；C、D：不同浓度吉西他滨对肺鳞癌增殖影响；E：微波热疗与吉西他滨不同序贯方式示意图；F、G：不同序贯方式对细胞增殖的影响。与对照组相比，^*^：*P* < 0.05，^**^：*P* < 0.01，^***^：*P* < 0.001；与吉西他滨组相比，^#^：*P* < 0.05，^##^：*P* < 0.01，^###^：*P* < 0.001。 The effects of microwave hyperthermia and gemcitabine on the proliferation of two lung squamous cell carcinoma cells. A, B: Effects of microwave hyperthermia at different temperatures and different times on the proliferation of lung squamous cell carcinoma cells; C, D: Effects of different concentrations of gemcitabine on the proliferation of lung squamous cell carcinoma cells; E: Diagrams of different sequential methods; F, G: Proliferation of the two cells in different sequential ways. Compared with the control group, ^*^: *P* < 0.05, ^**^: *P* < 0.01, ^***^: *P* < 0.001; compared with the gemcitabine group, ^#^: *P* < 0.05, ^##^: *P* < 0.01, ^###^: *P* < 0.001.

**1:** Veleriote法判断微波热疗与吉西他滨不同序贯方式联合对NCI-H1703细胞的影响（细胞增殖率%）（Mean±SD, *n*=3） The effect of microwave hyperthermia and gemcitabine to NCI-H1703 by Veleriote method (cell proliferation%)(Mean±SD, *n*=3)

NCI-H1703	Gem and MW	MW then Gem	Gem then MW
Gem	78.82±3.43	78.82±3.43	78.82±3.43
MW	74.45±8.03	74.45±8.03	74.45±8.03
MW combined with Gem	72.16±1.65	66.82±8.34	31.85±2.28
Predictive value	57.69±6.53	57.69±6.53	57.69±6.53
TYPE	Subadditive	Subadditive	Synergistic

**2:** Veleriote法判断微波热疗与吉西他滨不同序贯方式联合对NCI-H2170细胞的影响（细胞增殖率%）（Mean±SD, *n*=3） The effect of microwave hyperthermia and gemcitabine to NCI-H2170 by Veleriote method (cell proliferation%)(Mean±SD, *n*=3)

NCI-H2170	Gem and MW	MW then Gem	Gem then MW
Gem	73.29±2.50	73.29±2.50	73.29±2.50
MW	78.67±3.58	78.67±3.58	78.67±3.58
MW combined with Gem	64.93±5.13	52.05±2.11	24.25±4.30
Predictive value	50.99±1.72	50.99±1.72	50.99±1.72
TYPE	Subadditive	Subadditive	Synergistic

### 2.2 微波热疗联合吉西他滨抑制人肺鳞癌细胞的克隆形成

克隆形成试验统计结果显示：处理组的克隆形成数与对照组相比都有下降，且热化联合组下降的更为明显；热化联合组与单药化疗组相比，克隆形成数也明显下降(*P* < 0.001)。其中NCI-H1703细胞株对照组、微波热疗组、吉西他滨组和热化联合组的克隆形成数分别为(182.67±5.86)个、(143.67±5.67)个、(137.00±5.00)个、(17.67±1.53)个；NCI-H2170细胞株克隆形成数分别为(187.67±7.09)个、(141.00±4.58)个、(125.00±5.57)个、(24.67±2.52)个(图 2)。

**2:**
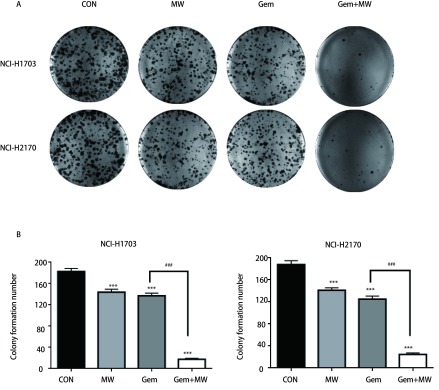
微波热疗联合吉西他滨抑制两株肺鳞癌细胞的克隆形成。A：克隆形成图片；B：克隆数目统计柱状分析。与对照组相比，^***^：*P* < 0.001；与吉西他滨组相比，^###^：*P* < 0.001。 Microwave hyperthermia in combination with gemcitabine inhibits the clonal formation of both cells. A: Pictures of clone formation; B: The number of statistical clones under the microscope. Compared with the control group, ^***^: *P* < 0.001; compared with the gemcitabine group, ^###^: *P* < 0.001.

### 2.3 微波热疗联合吉西他滨促进人肺鳞癌细胞发生Caspase-3依赖性凋亡

首先倒置显微镜下观察细胞形态变化：两株细胞的对照组其膜完整，细胞状态良好，边缘清晰；微波热疗组和吉西他滨组出现少量皱缩和空泡漂浮的细胞；热化联合组细胞皱缩脱落和空泡增多，其贴壁的细胞呈不规则生长，边缘模糊，折光率增高(图 3)。然后采用Annexin Ⅴ-FITC/PI双染法流式细胞术检测细胞凋亡，其凋亡结果显示：两株细胞热化联合组对比其他三组，细胞总凋亡率显著增加(*P* < 0.001)。其中NCI-H1703细胞株4组总凋亡率分别为(4.37±0.50)%、(16.57±5.68)%、(22.60±3.38)%、(45.63±1.33)%；NCI-H2170细胞株4组总凋亡率分别为(8.07±1.63)%、(13.47±3.84)%、(19.20±3.47)%、(65.01±6.07)%(图 4A)。接着采用Caspase-3、Caspase-8活性检测试剂检测各组细胞Caspase-3、Caspase-8酶活性，其结果显示：与其他三组相比，两株细胞热化联合组Caspase-3酶活性表达量明显上调(*P* < 0.01)(图 4Bb)；但与单独化疗组相比，两株细胞热化联合组Caspase-8酶活性表达量无明显变化，差异无统计学意义(*P* > 0.05)(图 4Bc)。为更进一步验证微波热疗联合吉西他滨是否促进人肺鳞癌细胞发生Caspase-3依赖性凋亡，采用CCK-8法检测加入AC-DEVD(Caspase-3抑制剂)后热化联合组细胞的增殖情况，其结果显示：加入AC-DEVD(Caspase-3抑制剂)后热化联合组细胞的增殖率高于热化联合组，其差异有统计学意义(*P* < 0.001)(图 4Bd)。

**3:**
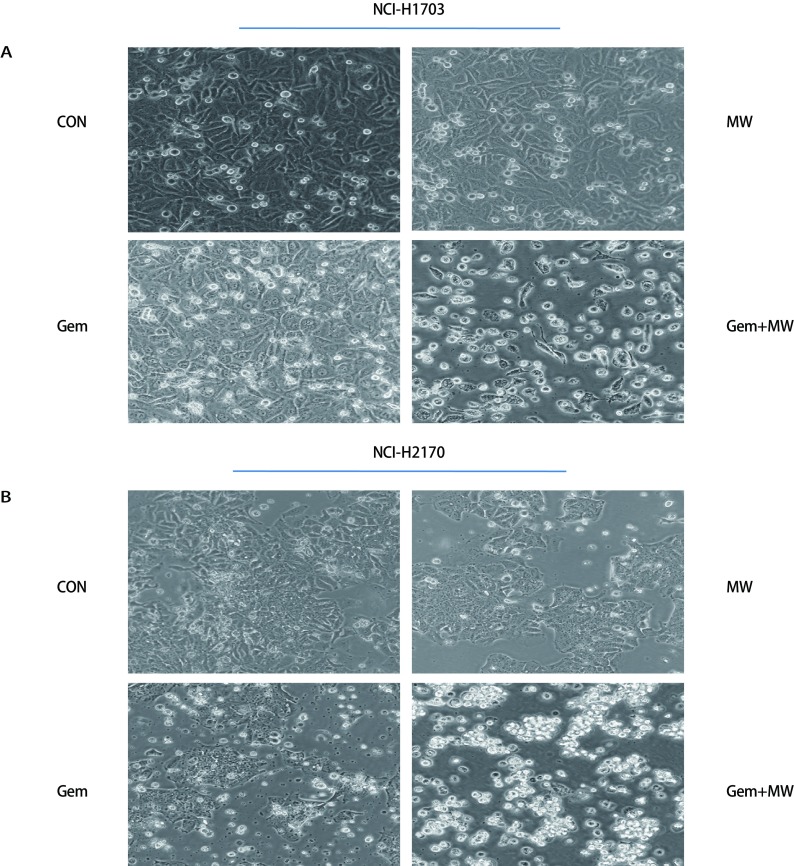
倒置显微镜下观察肺鳞癌细胞株NCI-H1703(A)和NCI-H2170(B)处理后的形态学变化(200×) The morphological changes of lung squamous carcinoma cell lines NCI-H1703 (A) and NCI-H2170 (B) were observed under an inverted microscope (200×)

**4:**
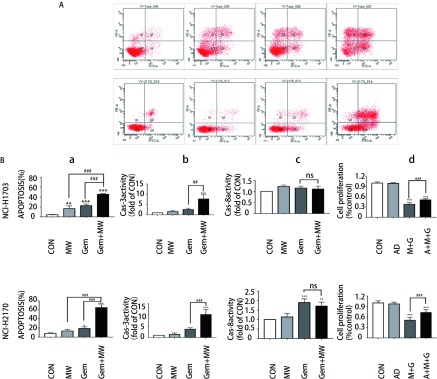
微波热疗联合吉西他滨诱导肺鳞癌细胞发生Caspase-3依赖性细胞凋亡。A：Annexin Ⅴ-FITC/PI双染法流式细胞术检测两株细胞的总凋亡率；B：a、b、c、d分别代表两株细胞总凋亡率、Caspase-3活性检测结果、Caspase-8活性检测结果及Caspase-3抑制剂(AC-DEVD)对肺鳞癌细胞增殖影响。与对照组相比，^*^：*P* < 0.05，^**^：*P* < 0.01，^***^：*P* < 0.001；与吉西他滨组相比，^###^：*P* < 0.001。 Microwave hyperthermia in combination with gemcitabine induces Caspase-3 dependent apoptosis in lung squamous cell carcinoma. A: Annexin Ⅴ-FITC/PI double staining flow cytometry was used to detect the total apoptosis rate of the two cells; B: a, b, c, d respectively represent the total apoptosis rate of two cells, Caspase-3 activity results, Caspase-8 activity results and Caspase-3 inhibitor (AC-DEVD) on lung squamous cell carcinoma cell proliferation. Compared with the control group, ^*^: *P* < 0.05, ^**^: *P* < 0.01, ^***^: *P* < 0.001; compared with the gemcitabine group, ^###^: *P* < 0.001.

### 2.4 微波热疗联合吉西他滨上调人肺鳞癌细胞中促凋亡相关蛋白的表达

两株细胞经微波热疗、吉西他滨以及微波热疗联合吉西他滨处理后应用Western blot检测各组细胞中凋亡相关蛋白表达情况，其结果显示：与对照组相比，两株细胞微波热疗联合吉西他滨组p53、Caspase-3、Cleaved-Caspase-3、Cleaved-PARP以及Bax蛋白明显上调(*P* < 0.001)，而PARP、Bcl-2蛋白明显下调(*P* < 0.001)；与吉西他滨组相比，微波热疗联合吉西他滨组p53、Caspase-3、Cleaved-Caspase-3、Cleaved-PARP蛋白明显上调(*P* < 0.05或*P* < 0.01)，而Bcl-2蛋白下调(*P* < 0.001)(图 5)。

**5:**
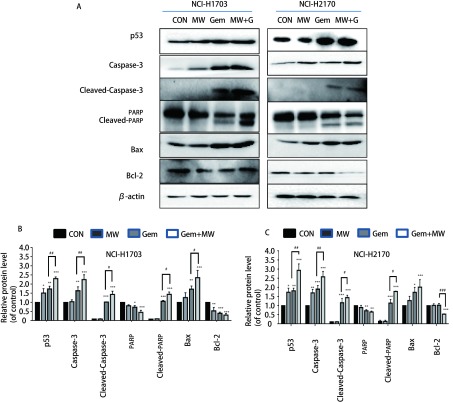
微波热疗联合吉西他滨对肺鳞癌细胞中的p53、Caspase-3、Cleaved-Caspase-3、PARP、Cleaved-PARP、Bax、Bcl-2表达的影响。A：蛋白电泳图；B、C：不同凋亡蛋白表达的柱状分析图。与对照组相比，^*^：*P* < 0.05，^**^：*P* < 0.01，^***^：*P* < 0.001；与吉西他滨组相比，^#^：*P* < 0.05，^##^：*P* < 0.01，^###^：*P* < 0.001。 The effects of microwave hyperthermia in combination with gemcitabine on the expressions of p53, Caspase-3, Cleaved-Caspase-3, PARP, Cleaved-PARP, Bax and Bcl-2 in lung squamous cell carcinoma cells. A: Protein electrophoresis; B, C: Columnar analysis of different apoptotic proteins. Compared with the control group, ^*^: *P* < 0.05, ^**^: *P* < 0.01, ^***^: *P* < 0.001; compared with the gemcitabine group, ^#^:*P* < 0.05, ^##^:*P* < 0.01, ^###^:*P* < 0.001.

## 3 讨论

时至今日，肺癌依旧占据着世界癌症发生率和死亡率的榜首，而我国肺癌发病率及死亡率也呈现出逐年上升的趋势。肺鳞癌作为肺癌常见的类型之一，发现时往往已处于晚期，无法进行手术治疗，其5年生存率很低^[12]^，因此研究肺鳞癌新的抗肿瘤联合疗法显得尤为重要。在本研究中，我们初步探讨了微波热疗联合吉西他滨在体外对肺鳞癌细胞株的增殖影响及诱导凋亡的机制。

热疗对化疗有明显的协同促进作用，热疗可以加快血流，增加血供，促进化疗药物在肿瘤局部的积聚和摄取，并降低肿瘤细胞的耐药性。多数化疗药物最大增敏作用出现在热化疗同步进行时^[15]^，但也有研究表明其最佳方案并不是热化同步：丹麦Overgaard利用C3H小鼠乳腺癌研究Etoposide、Ifosphamide与热疗的体内相互作用，结果显示热疗与Etoposide或Ifosphamide联用，对小鼠乳腺癌有明显的肿瘤抑制作用，加热前48 h注射Etoposide，治疗效果增加1.5倍，获得的最大效应时间间隔长于临床常规联合方案^[16]^。另有研究显示吉西他滨体内外试验其最大增敏作用出现在热化疗间隔前后24 h间^[17-20]^。本实验参考Satoko等^[18]^的序贯方法通过CCK-8法得出先化疗后微波热疗对肺鳞癌的增殖抑制具有协同作用，为了更进一步验证热化联合的协同作用我们进行了克隆形成实验，其结果与预期相符。

癌细胞活性的降低往往与抗肿瘤方法诱导其凋亡密切相关，Caspase半胱氨酸蛋白酶家族在细胞凋亡过程中发挥着重要的作用，处于级联反应下游的Caspase-3被认为是细胞凋亡的关键执行者，多种凋亡刺激信号的传递均汇聚于Caspase-3，它的活化代表着细胞凋亡进入不可逆阶段^[23]^。我们通过流式细胞术检测微波热疗和吉西他滨对两株肺鳞癌细胞凋亡的影响，结果发现微波热疗联合吉西他滨总凋亡率显著提高。接着通过Caspase-3、Caspase-8活性检测试验发现微波热疗联合吉西他滨组Caspase-3酶活性比单独化疗组明显提高，而Caspase-8无明显变化，这表明其诱导凋亡途径主要依赖于Caspase-3。当细胞发生凋亡时PARP即聚(ADP-核糖)聚合酶成为Caspase-3的重要切割底物，可加速凋亡进程^[24]^。目前研究得最多、最详细的凋亡途径是由p53蛋白介导的。p53主要可上调Bax蛋白的表达或者下调Bcl-2蛋白的表达，激活下游的促凋亡基因来诱发凋亡^[21, 22]^。已有研究表明先热疗(水浴)后吉西他滨治疗NSCLC可上调Caspase-3蛋白从而诱导其凋亡^[17]^，然而微波热疗联合化疗诱导癌细胞凋亡的机制研究甚少。本实验首次利用自主研发的微波热疗仪与吉西他滨联合探索其诱导肺鳞癌凋亡的机制，我们发现先吉西他滨后微波热疗作用于肺鳞癌细胞后与单独化疗相比，p53蛋白明显上调，并可发生Caspase-3的活化及PARP切割，表明其抑制肺鳞癌细胞的活性可能与诱导Caspase-3依赖性凋亡密切相关。

综上所述，本研究从细胞和分子层面初步探讨了微波热疗协同盐酸吉西他滨抑制肺鳞癌细胞NCI-H2170和NCI-H1703的增殖并诱导其凋亡，潜在的机制可能与启动P53通路，使PARP蛋白活化，激活Caspase-3依赖性凋亡途径诱导癌细胞发生凋亡，从而发挥其抑制癌细胞增殖的作用。但要进一步应用于临床，还有待于从动物层面进行实验验证。
